# Assessment of the Adjusted Clinical Groups system in Dutch primary care using electronic health records: a retrospective cross-sectional study

**DOI:** 10.1186/s12913-021-06222-9

**Published:** 2021-03-10

**Authors:** Shelley-Ann M. Girwar, Marta Fiocco, Stephen P. Sutch, Mattijs E. Numans, Marc A. Bruijnzeels

**Affiliations:** 1 Department of Public Health and Primary Care, Leiden University Medical Center, LUMC Campus The Hague, Turfmarkt 99, 2511 DP the Hague, the Netherlands; 2 Jan van Es Institute, Ede, the Netherlands; 3 Leiden University, Mathematical Institute, Leiden, the Netherlands; 4 Medical Statistics Department of Biomedical Data Science, Leiden University Medical Center, Leiden, the Netherlands; 5 Princess Maxima Center for Pediatric Oncology, Utrecht, the Netherlands; 6 Department of Health Policy and Management, Bloomberg School of Public Health Johns Hopkins University, Baltimore, MD USA

**Keywords:** ACG system, Risk stratification, General practice, Healthcare utilization, Health registry

## Abstract

**Background:**

Within the Dutch health care system the focus is shifting from a disease oriented approach to a more population based approach. Since every inhabitant in the Netherlands is registered with one general practice, this offers a unique possibility to perform Population Health Management analyses based on general practitioners’ (GP) registries. The Johns Hopkins Adjusted Clinical Groups (ACG) System is an internationally used method for predictive population analyses. The model categorizes individuals based on their complete health profile, taking into account age, gender, diagnoses and medication. However, the ACG system was developed with non-Dutch data. Consequently, for wider implementation in Dutch general practice, the system needs to be validated in the Dutch healthcare setting. In this paper we show the results of the first use of the ACG system on Dutch GP data. The aim of this study is to explore how well the ACG system can distinguish between different levels of GP healthcare utilization.

**Methods:**

To reach our aim, two variables of the ACG System, the Aggregated Diagnosis Groups (ADG) and the mutually exclusive ACG categories were explored. The population for this pilot analysis consisted of 23,618 persons listed with five participating general practices within one region in the Netherlands. ACG analyses were performed based on historical Electronic Health Records data from 2014 consisting of primary care diagnoses and pharmaceutical data. Logistic regression models were estimated and AUC’s were calculated to explore the diagnostic value of the models including ACGs and ADGs separately with GP healthcare utilization as the dependent variable. The dependent variable was categorized using four different cut-off points: zero, one, two and three visits per year.

**Results:**

The ACG and ADG models performed as well as models using International Classification of Primary Care chapters, regarding the association with GP utilization. AUC values were between 0.79 and 0.85. These models performed better than the base model (age and gender only) which showed AUC values between 0.64 and 0.71.

**Conclusion:**

The results of this study show that the ACG system is a useful tool to stratify Dutch primary care populations with GP healthcare utilization as the outcome variable.

**Supplementary Information:**

The online version contains supplementary material available at 10.1186/s12913-021-06222-9.

## Background

With rising health care utilization and costs, a shift from disease oriented to population based approaches is being advocated worldwide. With the upcoming need for improved organization and management of healthcare and the increasing possibilities of big data, strategies based on health registry analyses are becoming popular. One use of health registry data in population health management strategies is risk stratification. With risk stratification, differences in individual health risks can be screened for, and used to assign interventions to the population and individuals that will benefit the most. With rising pressure on medical services provided by general practitioners (GPs) in most European countries [1], primary care can benefit from proven advantages of risk stratification approaches, such as improved care management [2], resource allocation [3] and identification of sub-populations for tailored care interventions [4].

Despite the proven benefits of using risk stratification, especially in primary care, there is no evidence for application of internationally used risk stratification tools in Dutch primary care. Risk stratification approaches using Dutch GP registry data can be especially beneficial due to the gatekeeper’s function of Dutch GPs, providing the opportunity to overview a near total population.

Different tools for risk stratification are used worldwide, amongst which the Adjusted Clinical Groups (ACG) tool developed by the Johns Hopkins University. The ACG system is an internationally used tool for risk stratification on a generic level and is one of the most frequently used risk stratification tools in primary care. Evidence has also shown stronger statistical validity for the ACG compared with other risk stratification tools, regarding predictions of different healthcare utilization outcomes [5–7].

The ACG system uses registered diagnoses over a 12 month period, to assign individuals to one of 98 ACG categories, based on their healthcare profiles and expected health utilization [8]. ACG categories are based on combinations of diagnoses types. Registered diagnoses processed by the ACG system, can include the International Classification of Primary Care (ICPC) coded [9], a commonly used registration method for diagnoses in primary care [10].

In this study we explored the potential use of Johns Hopkins University ACG System in routine registration data extracted from Dutch primary care practices. The aim of this study is to explore how well the ACG system, compared to the 17 chapters of the ICPC coding system, can distinguish between different levels of GP healthcare utilization in Dutch general practice registries.

## Methods

### Study design and data

For this retrospective cross-sectional study, we used data from patients registered with one of the five participating GP practices during the whole of 2014 in Nijkerk, the Netherlands. Data for 30,596 patients over the year 2014extracted from the practices’ electronic health records.included age, gender, and coded healthcare procedures, diagnoses and pharmaceutical data. Diagnoses were registered as ICPC-1 diagnoses codes, as used in the Netherlands [11] and converted to ICPC-2 codes. Prescribed medication was registered as Anatomical Therapeutic Chemical (ATC) codes [12], GP visits were defined as all GP encounters, including physical and telephone consults and home visits by either GPs or nurse practitioners working at the GP practices.

From the original datasets 4289 cases were removed, due to corrupted patient identification numbers. Another 2689 cases belonging to three specific ACG categories, were left out of the analyses: *No Diagnosis or Only Unclassified Diagnosis* (*n* = 281), *Non-Users* (*n* = 2407) and *Invalid Age or Date of Birth* (*n* = 1). The final analyses were performed with data for 23,618 persons (77% of 30,596 registered people).

Data preparation and analyses were performed with IBM SPSS Statistics 24.

### ACG system software

We used the Johns Hopkins University’s ACG® System software 11. The ACG® System software 11 is a risk stratification tool, assigning each patient to one of the 98 mutually exclusive ACG categories. Assignment to ACG categories is based on combinations of diagnoses types. With the ACG system the diagnoses for each patient are grouped into 32 Aggregated Diagnosis Groups (ADGs), based on type of diagnoses rather than on specific diagnoses, i.e. specific ICPC codes. Individuals’ patterns of ADGs determine the assignment of patients to one of the 98 mutually exclusive ACG categories [8].

### Assessment of the ACG system

To assess the applicability of the ACG system in Dutch primary care, we looked at two aspects: face validity and model performance.

#### Face validity

According to Mosier [13] an important aspect of the testing of an instrument lies in the ‘consumer acceptance’. The first step in effective use of a test, is the actual selection for use and acceptance of the results. Mosier describes one of the translations of face validity as the appearance of validity: the test must appear valid in addition to the statistical validity. In this study we defined face validity as this appearance of validity described by Mosier [13].

We assessed the ACG system’s face validity by exploring the actual ACG categorization with regard to age. Face validity was assessed on recognition of multimorbidity in relation to age within ACG categories. The ACG categories are grouped according to the number of ADGs: one, two to three, four to five, six to nine and lastly ten plus ADGs.

#### Model performance

To investigate the impact of the ACG system in Dutch primary care, four different logistic regression models were estimated.

##### Dependent variable

The outcome variable, number of GP visits, was transformed into binary variables according to four definitions. According to the first definition, *no GP visits* was defined as *no utilization of care*, whereas *one or more GP visits* were defined as *utilization of care*. With the second definition, a distinction between *zero or one GP visit* and *two or more GP visits* was made. With the third definition, a distinction between *zero to two GP visits* and *three or more GP visits* was made. Accordingly, for the final definition the outcome was defined as a distinction between *zero to three* and *four or more GP visits*. The performance of each of these models was investigated.

##### Independent variables

In the null or base model only *age* as a continuous variable and gender were included as explanatory variables.

Model 1 included age, gender and ICPC chapters as independent variables. ICPC diagnosis codes are divided into 17 different chapters including *‘General and unspecified’*, ‘*Blood, blood forming organs, lymphatics, spleen’*, *‘Digestive’*, *‘Eye’*, *‘Ear’*, *‘Circulatory’*, *‘Musculoskeletal’*, *‘Neurological’*, *‘Psychological’*, *‘Respiratory’*, *‘Skin’*, *‘Endocrine metabolic and nutritional’*, *‘Urology’*, *‘Pregnancy, childbirth, family planning’*, *‘Female genital system and breast’*, *‘Male genital system’* and *‘Social problems’*. Different ICPC chapters can be registered to a single person. Therefore, the ICPC chapters were added to the model as 17 different dummy variables.

Model 2 included age, gender and ADG diagnoses as independent variables. As an individual can have more than one ADG, the 32 ADGs were added to the model as 32 dummy variables.

Model 3 included age, gender and mutually exclusive ACGs. Before estimating the logistic regression, the numbers of individuals in each ACG category were checked. Aggregation of some ACG categories was necessary due to categories with small numbers of individuals. In the Additional file 1 the aggregation of the original ACG categories is presented.

To select the best model, the performance of each logistic regression with outcome variable as defined above, was investigated. The Area Under the Curve (AUC) values were calculated for each model.

### Ethics approval and patients’ consent

The need for ethical approval was waived by the medical ethical committee of Leiden University Medical Center (CME - LUMC), the Netherlands.

Participants were not asked for their consent because we used routinely collected de-identified data.

## Results

### Population characteristics

**Fig. 1:**
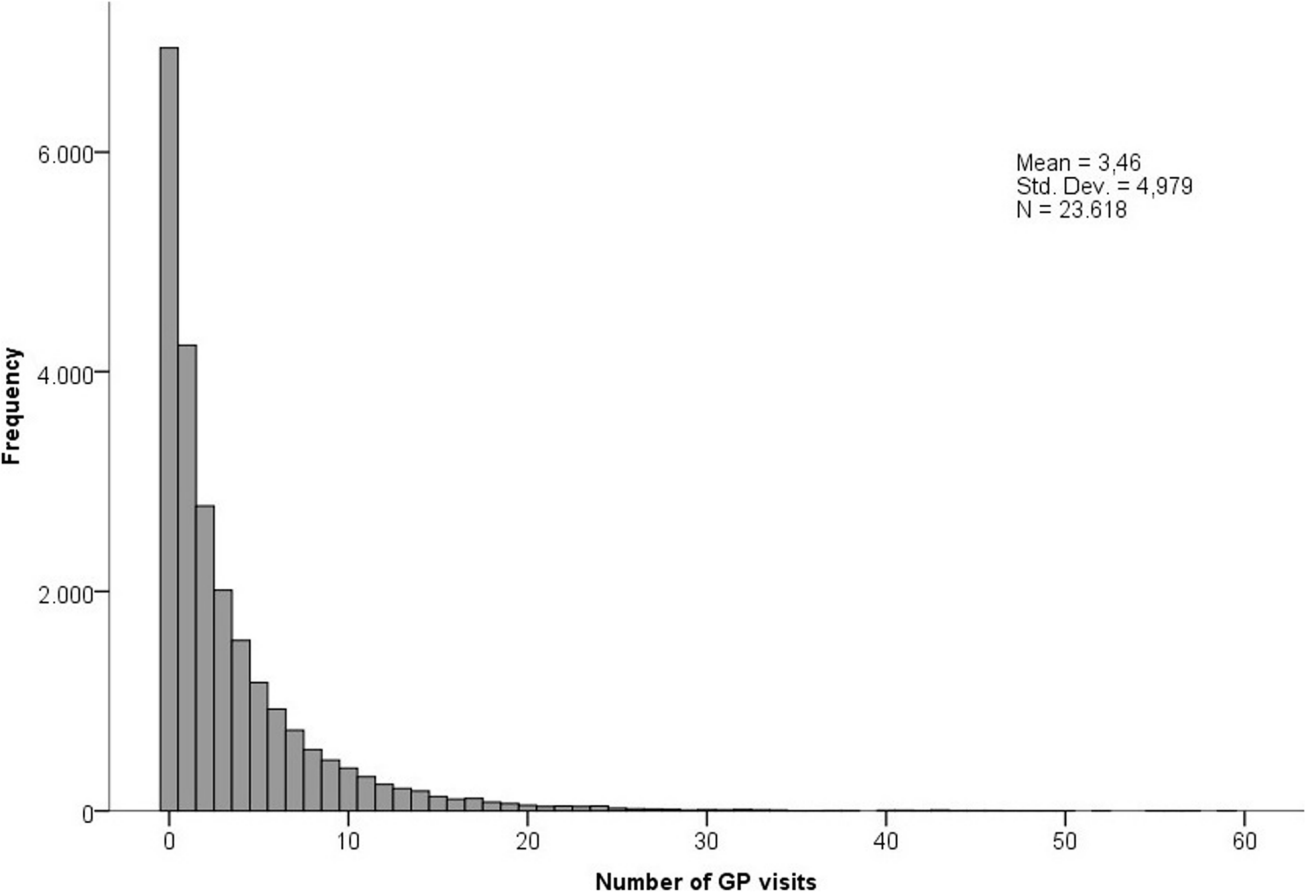
Distribution of the number of general practitioner (GP) visits within the study population

A total of 23,618 patients registered with a GP, were included in this study. 48.1% of the patients was male. The mean age of the included patients was 41.8 years old with a standard deviation of 22.2 years. 67.7% of the patients had at least one GP visit in 2014. The mean number of GP visits was 3.5 with a standard deviation of 5.0 and the maximum number of GP visits was 92. In Fig. 1 the distribution of the number of GP visits within the study population is presented. As expected, this is a skewed distribution, where most of the population has had zero or one GP visits.

**Fig. 2:**
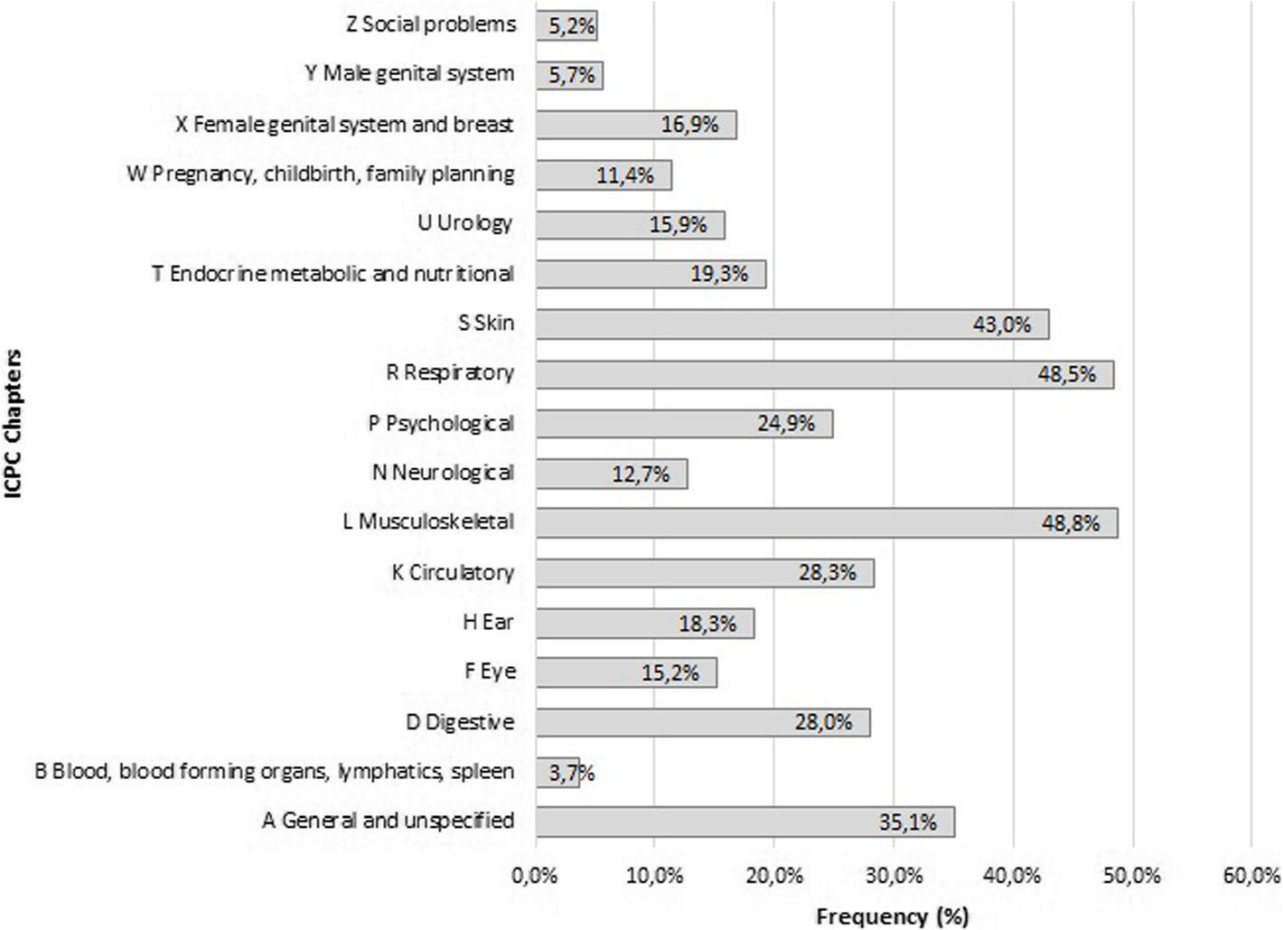
Overview of health problems within the study population according to the 17 main chapters of the International Classification of Primary Care (ICPC) coding system. ICPC chapters form the basis of the ICPC coding system

Figure 2 shows the health problems within the study population according to the 17 chapters of the ICPC registry system. The percentages of the study population with at least one diagnosis code corresponding to a specific ICPC chapter, are presented in the figure. ICPC chapters *Musculoskeletal* (L), *Respiratory* (R) and *Skin* (S) had the highest frequencies, with percentages between 43 and 49.

### Face validity of ACG categorization

**Fig. 3:**
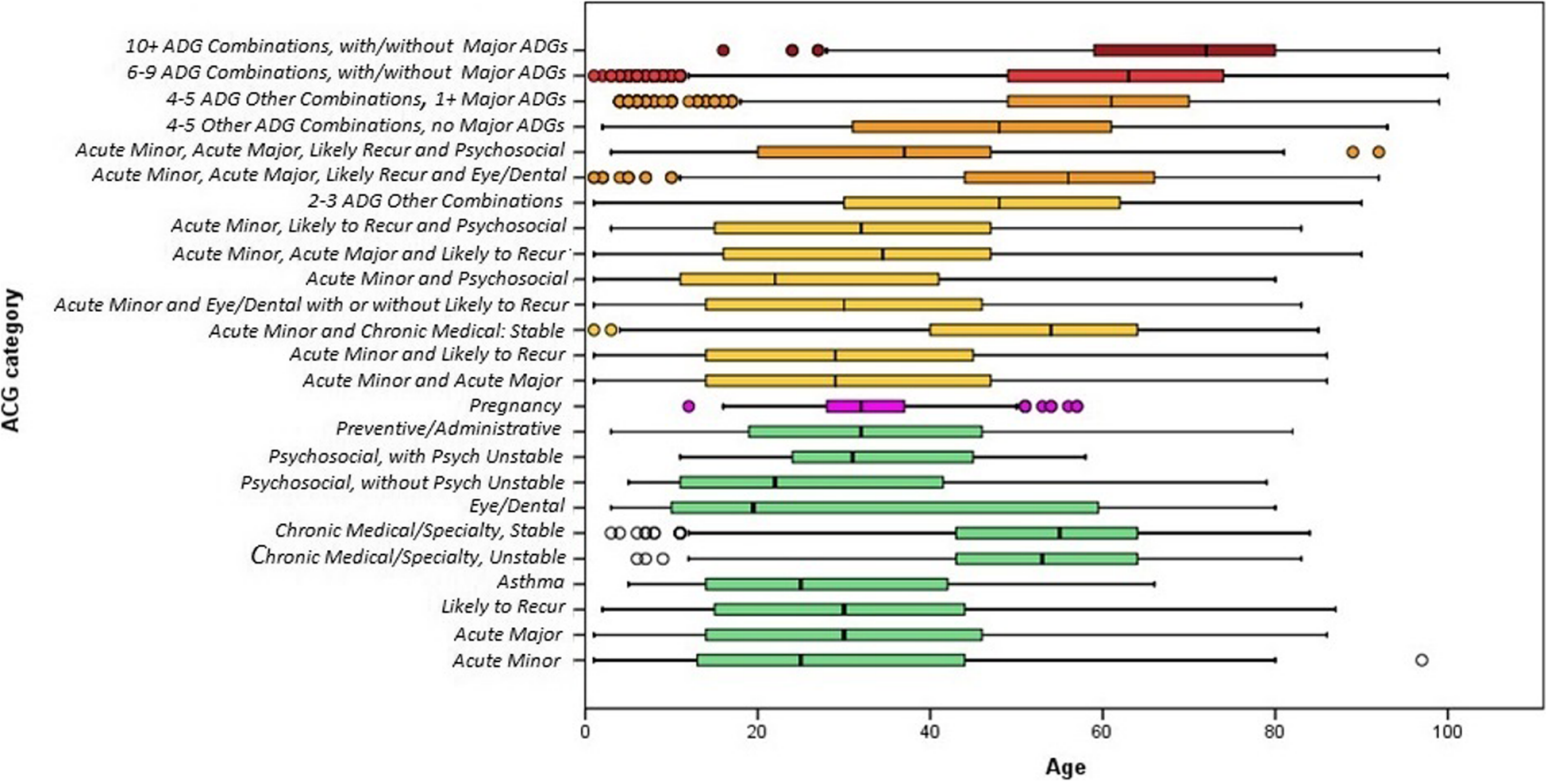
Age distribution per Adjusted Clinical Groups (ACG) category. ACG categories are a collapsed version of the original ACGs. Colors (with the exception of the pink one) correspond to the number of Aggregated Diagnostic Groups (ADGs): green = One ADG; pink = Pregnancy (all numbers of ADGs); yellow = 2–3 ADGs; orange = 4–5 ADGs; red =6–9 ADGs; dark red = 10+ ADGs

In Fig. 3 the distribution of age within each ACG category is presented with boxplots. Each group of ACGs corresponds with a different color, red being the highest numbers of ADGs. The figure shows that the number of ADGs gradually goes up with increasing age. Mean ages of the ACG categories with only one ADG *(green)* are mostly under 30. Exceptions are the ACG categories *Chronic medical: Stable* and *Eye/*Dental, which have mean values above 50. The mean age of ACGs with two to three ADGs *(yellow)* is mostly between 30 and 40, with the exception of ACG category *Acute Minor and Chronic Medical: Stable* (mean age of 50+). For three out of four of the ACG categories with four to five ADGs, the mean ages are between 50 and 62. However, the ACG category *Acute Minor/Acute Major/Likely Recur/Psychosocial* has a mean age of under 40. The ACG categories with six to nine ADGs have a mean age of around 63, whereas the mean age of ACG categories with ten or more ADGs is above 70. An extended overview of individuals from each ACG category, distributed over 10 year age bands, is presented in the Additional file 2.

### Model performance

To investigate the model performances, where the outcome variable utilization of GP was defined as discussed in the methods section, AUCs along with their confidence intervals were computed.

Table 1 displays the model performances for each of the four different definitions of the outcome GP utilization. As seen in the table, model 1 and 2 perform well with AUC values between 0.79 and 0.85. They slightly perform better than model 3 with AUC values between 0.77 and 0.83. All three models outperform the null model with AUC values between 0.63 and 0.71. For all independent variables, odds ratios along with their 95% confidence intervals, are shown in Additional files 3, 4 and 5.

**Table 1:** Model performances quantified by the Area Under the ROC Curve (AUC) values along with the 95% confidence intervals (CI)

Area Under the ROC Curve (95% Confidence Interval)				
Outcome	Null model	Model 1	Model 2	Model 3
**0 vs. > = 1 GP visits**	0.638 (0.630–0.645)	0.787 (0.781–0.793)	0.793 (0.787–0.799)	0.774 (0.768–0.780)
**0–1 vs. > = 2 GP visits**	0.675 (0.668–0.681)	0.816 (0.810–0.821)	0.818 (0.812–0.823)	0.799 (0.794–0.805)
**0–2 vs. > = 3 GP visits**	0.693 (0.686–0.700)	0.833 (0.828–0.838)	0.832 (0.828–0.837)	0.814 (0.809–0.819)
**0–3 vs. > = 4 GP visits**	0.711 (0.704–0.718)	0.848 (0.842–0.853)	0.848 (0.842–0.853)	0.829 (0.824–0.834)

‘Outcome’ is based on the four definitions of the outcome general practice (GP) healthcare utilization

## Discussion

The results of this study suggest that the ACG system can be applied to Dutch primary care data, when regarding both face validity and model performance. With regard to the face validity, it can be concluded that the assignment of ACG categories is as expected: the ACG categories which indicate higher multimorbidity and thus higher expected care burden, are found amongst older patients. With respect to model performance, results showed that distinctions between the different levels of GP healthcare utilization can be made with the ACG system. The ACG and ADG categories, as well as the ICPC chapters (the commonly used primary care coding system), are highly associated with GP utilization. However, the ACG system is at patient level and provides a variety of other risk stratification variables, such as multimorbidity measures, risks of hospitalization and high costs, making the use of the ACG as risk stratification tool a good addition to the use of the ICPC coding system.

Comparison of the results of this study to previous research is challenging, as most previous studies investigating the association of the ACG system with continuous utilization outcome measures. Some previous studies were carried out on dichotomous variables however and showed C-statistics and AUC values between 0.73 and 0.82 for the ACG as predictor for hospitalization [5, 6, 14]. In addition, the study by Haas et al. presented C-statistics of 0.67 for emergency department visitation and 0.76 for top 10% healthcare costs [5].

Adding to the above mentioned studies, this study suggests that the ACG system is applicable in primary care. Analyzing primary care data in such a manner is of great importance for the understanding of efficiency of healthcare systems that are under increased physical and financial pressure. A study by Sibley et al. showed that administrative data can be used to determine morbidity burden, an important indicator for future care utilization [15]. Kristensen and colleagues assessed the use of the ACG system as a morbidity based casemix adjustment system amongst type 2 diabetes patients in order to allocate resources according to degree of co-morbidity [3]. They stated that the Danish healthcare system, which is based on fee for service incentives, would profit from a morbidity based casemix adjustment system. The ACG has also proven to be effective for identifying inequities in healthcare utilization by Shadmi et al. [7]. Identifying inequities is the first step towards minimizing unwarranted care gaps. With risk stratification tools such as the ACG, case finding for inclusion in population-level interventions can be performed in more health systems worldwide. A study by Soto-Gordoa used risk stratification to select cases for a patient-centered intervention for multimorbid patients with the goal to lower hospitalization. The approach avoided 9 % of hospitalization when cases were selected with the ACG tool [4].

With our study, a first step towards validation of the ACG system, a tool to shift from disease oriented to population based approaches, is revealed for use in the Netherlands. This is opening up a variety of opportunities to reorganize and manage Dutch primary care in an efficient way.

Although the ACG seems an excellent tool to be used in the Netherlands, local adjustment of the software is of eminent importance. A limitation of this study might be the availability of only GP data (without, for example, hospital and mental health care data), forcing us to restrict healthcare utilization outcomes to GP visits, whereas healthcare utilization may be better defined as a total overview of healthcare use. With our research we were not able to explore other types of healthcare utilization, for example defined by total healthcare costs or more costly types of healthcare utilization such as hospitalization and emergency department visitation. Consequently, a full adjustment of the ACG system for use with Dutch data was not possible yet. Further exploration of the ACG system with the use of different data sources will follow.

Moreover, the quality of data needs to be considered. For this study, routine data from GP registries were used**.** Risk stratification with routinely collected primary care data is an easy and practical way to perform risk stratification on a large scale. Data quality for risk stratification purposes can be improved and strengthened by linkage with different data sources such as hospital and social care registries. The exclusion of social data, such as ethnicity and underlying socio-economic variables, is another limitation of this study. Ethnicity and even more the underlying socio-economic aspect thereof, may have important aspects on patient’s health profiles. The addition of social variables and thus more complete patient profiles are of added value in risk stratification approaches. However, we were unable to include these data in our models, as they were not available in the GP data.

### Policy implication

Even though the use of the ACG system typically recommends the use of both primary care and hospital care data, this study shows that the ACG is very promising with the use of solely primary care data, especially in a primary care system with mandatory GP listing. With the possibility of applying risk stratification tools to such primary care based healthcare systems, without the need to link data from different sectors, the information security issues can be avoided. Patients’ personal information is already available to GP’s for optimal caregiving purposes.

With addition of other data sources on individual patient’s level, regulations need to be considered to allow the linkage of personal data. As the value of adding hospital data is still to be explored, further research on both content-specific and regulatory aspects is desirable.

Altogether, applications such as the ACG, are very promising for healthcare systems, as their ability to predict future health utilization can be beneficial for person-tailored health intervention strategies, such as screenings for care management interventions, as well as local, regional or even nationwide healthcare management.

### Further research

Before applying the ACG system in Dutch primary care, further research is required. This study showed associations between just two components of the ACG system, the ADG and ACG categories, and GP visitation. Risk scores, for example, for future hospitalization and total healthcare costs were outside the scope of this study. To justify the use of the ACG system as risk stratification tool in Dutch primary care, studies validating the ACG risk scores should be conducted. In addition, the ACG models need to be adjusted and improved for use with Dutch primary care data.

## Conclusions

This study showed that the ACG is applicable as risk stratification tool in Dutch primary care using routinely registered data from general practitioners’ registries. The ACG system yields good results compared to the traditional ICPC classification. Country specific adjustments in the classification and validation of specific risks are necessary.

## Supplementary Information

**Additional file 1.** Aggregation of the original ACG categories for logistic regression model 3.

**Additional file 2.** Extended overview of individuals from each ACG category, distributed over 10 year age bands.

**Additional file 3.** Odds Ratio with 95% confidence intervals for dependent variables in model 1 with the second definition for GP utilization.

**Additional file 4.** Odds Ratio with 95% confidence intervals for dependent variables in model 2 with the second definition for GP utilization.

**Additional file 5.** Odds Ratio with 95% confidence intervals for dependent variables in model 3 with the second definition for GP utilization.

## Data Availability

Restrictions apply to the availability of the data that support the findings of this study. As data contain personal health information of individuals, data were strictly used under license for the current study, and are therefore not publicly available.

## Abbreviations

ACG: Adjusted Clinical Groups
ADG: Aggregated Diagnosis Group
ATC: Anatomical Therapeutic Chemical codes
AUC: Area Under the Curve
GP: General practitioner
ICPC: International Classification of Primary Care
ICPC-1: International Classification of Primary Care, version 1
